# Mechanistic role of a disease-associated genetic variant within the ADAM33 asthma susceptibility gene

**DOI:** 10.1186/1471-2350-8-46

**Published:** 2007-07-17

**Authors:** Richard G Del Mastro, Laura Turenne, Heidi Giese, Tim P Keith, Paul Van Eerdewegh, Klaus JW May, Randall D Little

**Affiliations:** 1 Molecular Therapeutics Division, AmberGen Incorporated, Waltham, Massachusetts 02453, USA; 2 Cambridge, MA 02139, USA; 3 Genomatix Software GmbH, D-80335 Munich, Germany; 4 Genizon BioSciences, Quebec, H4T 2C7, Canada

## Abstract

**Background:**

ADAM33 has been identified as an asthma-associated gene in an out-bred population. Genetic studies suggested that the functional role of this metalloprotease was in airway remodeling. However, the mechanistic roles of the disease-associated SNPs have yet to be elucidated especially in the context of the pathophysiology of asthma. One disease-associated SNP, BC+1, which resides in intron BC toward the 5' end of ADAM33, is highly associated with the disease.

**Methods:**

The region surrounding this genetic variant was cloned into a model system to determine if there is a regulatory element within this intron that influences transcription.

**Results:**

The BC+1 protective allele did not impose any affect on the transcription of the reporter gene. However, the at-risk allele enforced such a repressive affect on the promoter that no protein product from the reporter gene was detected. These results indicated that there exists within intron BC a regulatory element that acts as a repressor for gene expression. Moreover, since SNP BC+1 is a common genetic variant, this region may interact with other undefined regulatory elements within ADAM33 to provide a rheostat effect, which modulates pre-mRNA processing. Thus, SNP BC+1 may have an important role in the modulation of ADAM33 gene expression.

**Conclusion:**

These data provide for the first time a functional role for a disease-associated SNP in ADAM33 and begin to shed light on the deregulation of this gene in the pathophysiology of asthma.

## Background

Over the past two decades, the paradigm for the identification of genes that cause monogenic diseases has proven to be extremely successful. Analytical and experimental tools that employ family-based linkage methods to scan the genome have identified major genes with causative mutations. These monogenic diseases are rare in nature but the disruption that is introduced into a single gene produces a large phenotypic effect. The application of the same tools to common disorders has proven to be far less lucrative. This is due to the complexity of polygenic disorders where the contributing effect of a single gene is reduced [1]. However, improved analytical methodologies that augment the power of association studies have revealed the location of elusive genes that underlie complex disease phenotypes [2-4]. Linkage disequilibrium mapping in combination with the discovery of millions of single nucleotide polymorphisms (SNPs) have led to the development of high resolution haplotype maps that can be utilized to discover disease-associated loci [5-7]. This has led to a resurgence of the positional cloning paradigm and the identification of disease-susceptibility genes.

Using these approaches, the first asthma susceptibility gene, ADAM33 (A Disintegrin And Metalloprotease 33), was discovered in an outbred population. Linkage analysis was conducted on 460 Caucasian affected sib-pair families from UK and US populations. Significant linkage to asthma and bronchial hyperresponsiveness was identified in close proximity to the tip of the p-arm of chromosome 20. A subset of 130 unrelated asthma cases, which showed significant evidence of linkage to this region, and 217 unrelated controls were utilized in a case-control study on 135 SNPs that fell within the 90% confidence interval. Multiple SNPs and SNP pairs that were typed in ADAM33 were found to be associated with asthma and bronchial hyperresponsiveness [8]. Replication studies in diverse ethnic asthma populations reproduced the original work identifying multiple SNPs within the gene having association with asthma and its sub-phenotypes [9-20]. While these genetic studies have identified ADAM33 alleles that are associated with an asthma phenotype, the mechanistic role of the SNPs in the development of the disease symptoms has yet to be determined.

The locations of the ADAM33 disease-associated SNPs are within the coding and 3'untranslated regions as well as deep within introns [8]. While it is not always immediately evident as to the affect of a single nucleotide change on the function of the protein, several SNPs within the exons were found to introduce amino acid changes and provided some insight as to how they could disrupt the protein molecule [21]. Other exonic SNPs were found to be synonymous and cast little information as to their disruptive traits. The same was observed for the deep intronic and 3'UTR variants [8]. However, studies in recent years on the mechanistic role of genomic variants, whether they reside within introns or exons of a gene, have demonstrated that they can disrupt pre-mRNA splicing. The consequence of such a disruption is to induce exon skipping, enhance the use of cryptic splice sites and alter the ratio of alternatively spliced isoforms. Such perturbations have been shown to be the cause of various human disease phenotypes [22-27]. Furthermore, studies have shown that the promoter of a gene plays a role in exon selection as well as dictating the levels of transcription, indicating that pre-mRNA splicing is a complex set of events that can be perturbed by single or multiple genetic variants [28].

**Figure 1:**
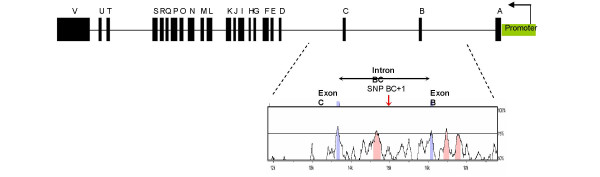
**A schematic showing the intron-exon structure of ADAM33 and the location of CNSs toward the 5' end of the gene**. The results are a comparison of human ADAM33 intron BC and the flanking sequence with the syntenic region of the mouse gene. The x axis corresponds to the human reference sequence (GenBank accession no: AF466288) and the y axis corresponds to the percent identity for a sliding window of 100 bp. Exons are labeled in blue and show high homology between the two species. Intronic regions >75% identical over at least 100 bp are shown in pink where there is a CNS region that is in close proximity to SNP BC+1 and two others that reside in intron AB. SNP BC+1 lies within a CNS that is >60% similar to the mouse. The promoter is in green showing the direction of transcription.

Determining the mechanistic role of disease-associated SNPs has proven to be challenging [29]. In ADAM33 there are several SNPs that are associated with the asthma phenotype. One SNP, BC+1, has been shown to have one of the highest associations with asthma (p = 0.0027) [30]. This genetic variant is located toward the 5' end of the ADAM33 gene deep within intron BC. In addition, comparative sequence analysis of this region with the mouse ADAM33 intron BC and the surrounding area has identified regions that are highly conserved between the two species [31]. This would suggest that there are conserved non-coding sequences (CNS) in this region of the gene that may contain regulatory elements. Furthermore, SNP BC+1 appears to be located within a CNS as well as in close proximity to other conserved regions (Fig. 1). We present data which shows for the first time that the genetic variant, SNP BC+1, has the functional capacity to disrupt transcription and lies within a critical region of ADAM33. Understanding the mechanistic role of this disease-associated SNP would begin to provide some insight into the effect of other genetic variants in this gene and how they may contribute to the asthma phenotype.

## Methods

### Cell culture

SV40 transformed lung fibroblasts (WI-38) (American Type Cultue Colection, Manassas, VA) were maintained in Eagle MEM (Invitrogen Corporation, Carlsbad, CA), 10% fetal calf serum (Invitrogen Corporation, Carlsbad, CA) and supplemented with 5 mM L-glutamine (Invitrogen Corporation, Carlsbad, CA) and 1.0 mM sodium pyruvate (Invitrogen Corporation, Carlsbad, CA). The cells were grown in T75 flasks until confluent. After this 5 × 10^6 ^cells were aliquoted into 6-well culture plates for transfection, in triplicate, of the constructs and controls in triplicate.

### Cloning intron BC

Six fragments of varying sizes from intron BC (4 spanned the SNP and 2 flanked it, which served as internal controls) (Fig. 1) were PCR amplified from BAC RP11-1098L22 (GenBank Accession no. AF466288) (Invitrogen Corporation, Carlsbad, CA). All primers were designed with 6T's (to allow efficient digestion) followed by a NheI (forward) and a BglII (reverse) restriction site at the 5' end (underlined). This was then proceeded by ADAM33 18 to 21 nucleotides of the ADAM33 intron BC sequence.

Construct #1/#4/#5 Fwd: TTTTTTGCTAGCGTTGACCAGAACATGTGACC

Construct #2/#6 Fwd: TTTTTTGCTAGCGCCATGAAGGCTGAGAGGC

Construct #3 Fwd: TTTTTTGCTAGCGGCAAGGACCATCTGCTCC

Construct #1/#2/#3 Rev: TTTTTTAGATCTGCTCCGACACCTGCTTCAC

Construct #4 #6 Rev: TTTTTTAGATCTGGAGCAGATGGTCCTTGCC

Construct #5 Rev TTTTTTAGATCTGCCTCTCAGCCTTCATGGC

The fragments were amplified by PCR using the Phusion High Fidelity DNA polymerase using conditions recommended by the manufacturer (New England Biolabs, Beverly, MA). PCR fragments were verified by gel electrophoresis, digested with NheI and BglII, gel purified with Qiaex II (Qiagen, Valencia, CA) and cloned into the multiple cloning site of the pSEAP2-control vector from the BD Great EscAPe SEAP Reporter System 3 kit (BD Biosciences, San Jose, CA). The vector contained a reporter gene, alkaline phosphatase, whose protein product is secreted into the conditioned medium and used to quantitate the functional effect of the alleles. Sequence analysis of the six clones was performed (Agencourt Biosciences, Beverly, MA) to ensure that no alterations had occurred during the PCR and cloning processes. The 4 clones that spanned the SNP BC+1 possessed the protective allele (A).

### Site directed mutagenesis

Quick Change Site-Directed Mutagenesis Kit (Stratagene, La Jolla, CA) was utilized to change the nucleotide at SNP BC+1 from an A (protective) to a G (at-risk) in Constructs #1, #2, #4 and #6 following the manufacturer's recommendations. All clones were sequenced (Agencourt Biosciences, Beverly, MA) to confirm the nucleotide change and the remainder of the sequence was inspected to ensure that no other alterations had occurred during the site directed mutagenesis process.

### Quantitation of the BC+1 alleles

Each of the 10 constructs were co-transfected in triplicate with pcDNA3.1His/LacZ (Invitrogen Corporation, Carlsbad, CA) into the WI-38 cell line using LF2000 (Invitrogen Corporation, Carlsbad, CA) according to manufacturers recommendations. The pcDNA3.1His/LacZ vector (Invitrogen Corporation, Carlsbad, CA) was utilized to normalize for transfection efficiency. Five hours after transfection the media was removed and replaced with OptiMem (Invitrogen Corporation, Carlsbad, CA). Forty-eight hours post transfection 200 μl of conditioned medium was removed from each well and transferred to 1.5 ml eppendorf tubes. The supernatant was clarified by centrifugation at 12000 × g for 1 minute and 15 μl was transferred to a luminometer plate. The amount of alkaline phosphatase protein present in the conditioned medium was measured using the BD Great EscAPe SEAP Chemiluminescence (BD Biosciences, Palo Alto, CA) kit and a Wallace Victor V luminometer following the manufacturer's recommendations. Transfection efficiency of the pcDNA3.1His/LacZ was measured using the Beta Galactosidase Assay Kit (Invitrogen Corporation, Carlsbad, CA) following the manufacturer's recommendations.

## Results

### The at-risk allele of BC+1 influences transcription

We hypothesized that a region within intron BC of the ADAM33 may have the capability to influence the promoter's ability to set the levels of transcription. If so then based on its location relative to the promoter this regulatory region would need to loop back on itself. Thus, the regulatory region in intron BC would position itself with the promoter and both would work in concert to produce the appropriate transcript levels for that cell type [28]. If this would be the case then the protective (Pr) or at-risk (Ar) allele may alter the structure of the regulatory region, which may consequently have an effect on its interaction with the promoter.

**Figure 2:**
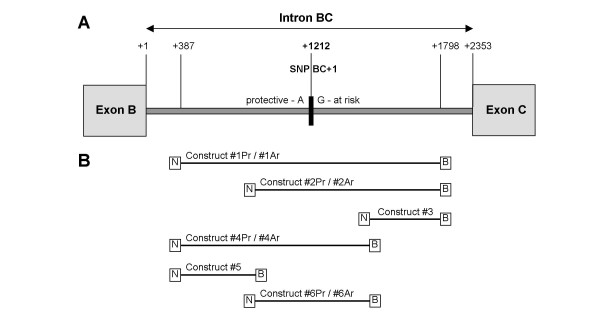
**Analysis of SNP BC+1 within intron BC of ADAM33**. **(A) **Schematic showing the location of SNP BC+1 within intron BC and the protective (Pr) and at-risk (Ar) alleles A and G respectively. **(B) **Schematic defining the constructs generated to analyze the function of the two alleles A and G. The PCR generated fragments were cloned into the multiple cloning site at the NheI (N) and BglII (B) restriction sites of the pSEAP2 Basic vector.

The genetic variant SNP BC+1 (NCBI SNP database accession number: rs487377) [55] is located at the 5'end of the ADAM33 gene within intron BC at nucleotide 1212 between exons B and C (Fig. 2A). The polymorphism is an A to G. The A allele has been shown to be protective and the G at-risk for asthma [30]. Six overlapping fragments from intron BC were generated by PCR using BAC RP11-1098L22 as the template. Four of the six fragments spanned the polymorphic site and the remaining two flanked the region. The six fragments were cloned into the pSEAP 2-control vector upstream of a SV40 promoter and an alkaline phosphatase reporter gene, which created a basic model that simulated a simple contextual arrangement of the putative intron BC regulator with a promoter. The 4 clones that spanned the polymorphic site possessed the protective (Pr) allele A. Site directed mutagenesis was employed to replace the A nucleotide and introduce the at-risk (Ar) allele G into this site (Fig. 2B). The 10 clones were transformed into the WI-38 cell line and the amount of alkaline phosphatase protein secreted into the medium was measured for each construct to quantitate the effects of the protective and at-risk allele on transcription of the reporter gene.

**Figure 3:**
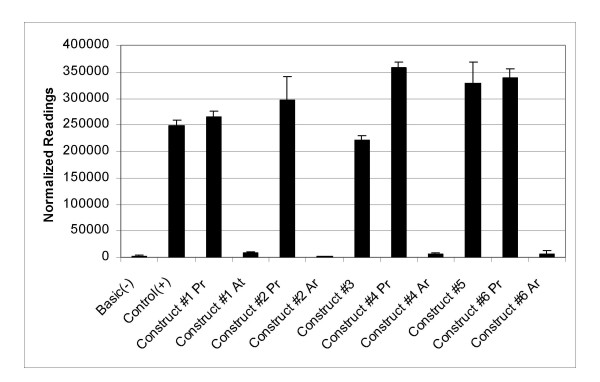
**Results of quantitating the amount of secreted alkaline phosphatase protein in the medium**. The luminescence readings taken from the alkaline phospahatse measurements were normalized against the measured amount of beta galactosidase to obtain the normalized readings. All constructs that contained the Ar allele showed normalized readings similar to the Basic (-) control, which did not possess the SV40 promoter. The opposite result was observed for the constructs that contained the Pr allele. Pr allele constructs showed normalized readings similar to the Control (+), which possessed the SV40 promoter and the alkaline phosphatase reporter gene.

The constructs that contained the protective allele A, Construct #1 Pr, #2 Pr, #4 Pr and #6 Pr, produced a measurable amount of alkaline phosphate. These levels were similar to those produced by the control (+), which contains only the SV40 promoter that drives the transcription of the alkaline phosphatase reporter gene (Fig. 3). This result was also observed in the two constructs, Construct #3 and #5, which flanked the SNP BC+1 region. Conversely, the constructs that contained the at-risk allele G, (Construct #1 At, #2 At, #4 At and #6 At) did not produce any measurable secreted alkaline phosphatase protein (Fig. 3). This was similar to the negative control, Basic (-), which only contains the reporter gene without the SV40 promoter. These results indicated that the genetic variant at nucleotide position 1212 within intron BC possess a functional capacity, whose mechanism of action is to interact with the promoter. Consequently, depending upon the polymorphism its affect can either have no influence on transcription, the protective allele A, or it can abrogate the SV40 promoter, the at-risk allele G, to repress transcription of the reporter gene and diminish the production of the alkaline phosphatase protein.

### Analysis of the sequence surrounding the SNP BC+1

To determine whether any regulatory regions reside within the locale of SNP BC+1 computational tools were applied to the DNA sequence within intron BC, which scanned for *cis*-acting element/s. The sequence surrounding the SNP BC+1 was examined for the presence of transcription factor (TF) binding sites using ElDorado (Genomatix Software GmbH), which graphically displays all the promoter modules within genes using proprietary software and public domain information. Fifty one nucleotides, 25 nucleotides to the left and to the right of SNP BC+1, were analyzed for the presence of TFs. When the protective allele A was present in the sequence, a region that shared significant similarity to the NFkappaB p50 sub-unit binding site was identified. However, when the procedure was performed on the same stretch of DNA, which contained the at-risk allele G a different result was obtained. The region that shared the homology with NFkappaB p50 sub-unit was eliminated. NFkappaB is a ubiquitous transcription factor formed by various homo and heterodimers of the NFkappaB family and regulates genes involved in immune and inflammatory responses [32-34]. These computational studies of the genomic region surrounding SNP BC+1 indicated that nucleotide position 1212 within intron BC has the potential to either introduce or abolish a functional regulatory element.

## Discussion

Currently, 42 disease-associated SNPs have been identified within the asthma-associated ADAM33 gene [30]. While several SNPs have been shown to be associated with asthma or its sub-phenotypes none have been identified to possess a functional role that would explain how these genetic variants could contribute to the disease. This study demonstrates for the first time that an ADAM33 asthma-associated SNP, BC+1, does have functional consequences. Through the use of an *in vitro *model system, we have demonstrated that the at-risk allele of SNP BC+1 has an assertive influence on the promoter causing a disruption to the mechanisms that drive transcription. To understand the implication of this result in the context of ADAM33 regulation one needs to view these data with regards to the spatial arrangement of the regulatory region within the gene. The BC+1 SNP and the surrounding region were placed upstream of the SV40 promoter in the *in vitro *construct, which generated the observed result. However, this region is located downstream of the ADAM33 promoter in an intron that is flanked by an exon. In addition, further upstream of SNP BC+1 there is intron AB and exon A, which contains the 5'UTR. Thus the contextual arrangement of the SNP BC+1 region is vastly different in the ADAM33 gene compared with our *in vitro *model system, which possess an intronless reporter gene. Yet the model system demonstrates that SNP BC+1 lies within a regulatory region that interacts with the promoter and is capable of suppressing its role to transcribe the reporter gene when the at-risk allele G is in the polymorphic site. Therefore, it is plausible that this regulatory region, within intron BC, may work closely with the ADAM33 promoter and perhaps other as yet undefined regulatory regions within the gene to influence expression levels, which are appropriate for a particular cell-type.

Studies have demonstrated that the promoter of a gene can work in concert with other *cis*-acting elements, which couple both transcription and pre-mRNA splicing [35-37]. In this present study we provide experimental evidence to suggest that the nucleotide position 1212 within intron BC has a functional role, which is coupled with the promoter. One allele, the at-risk, appears to generate a repressor element suppressing expression of the reporter gene whereas the other, the protective does not cause any perturbation in protein levels. However, *in vivo *the mechanism of action of the SNP may behave like a rheostat when combined with other regulatory regions within ADAM33. As the interactions of these regulatory regions can be complex, a perturbation that disrupts the modulation of pre-mRNA processing could be subtle yet acutely felt, in particular in the bronchial smooth muscle cells and lung fibroblasts, where ADAM33 has been shown to be expressed [8,38]. Disruptions to the normal modulation of expression levels and splicing patterns that have been introduced by genetic variants can be a cause or modifier of human diseases [23]. Consequently, these subtle effects produced by polymorphic sites such as the one at BC+1 could be contributors to the pathophysiology of asthma.

The processing of ADAM33 pre-mRNAs may be influenced by a variety of control elements including the region around BC+1. This might explain the numerous alternative splice variants that have been detected [39,40]. In addition ADAMs, which play an important part in the regulation of cell signaling through cytokine and growth factor shedding are tightly regulated [41]. ADAM33 is no exception. Studies have demonstrated that only 10% of the ADAM33 protein is localized to the cell surface. The remainder is packaged into the endoplasmic reticulum and the proximal Golgi and utilized only under appropriate conditions [42]. The data presented in this paper would point to the genetic variant within intron BC as one possible cause for a disruption in the tightly regulated processing of ADAM33. The ramification of such a perturbation would reverberate through to the protein level where the isoform ratios could be altered and as such may augment or diminish the cell signaling mechanisms leading to a disease outcome [43,44].

**Figure 4:**
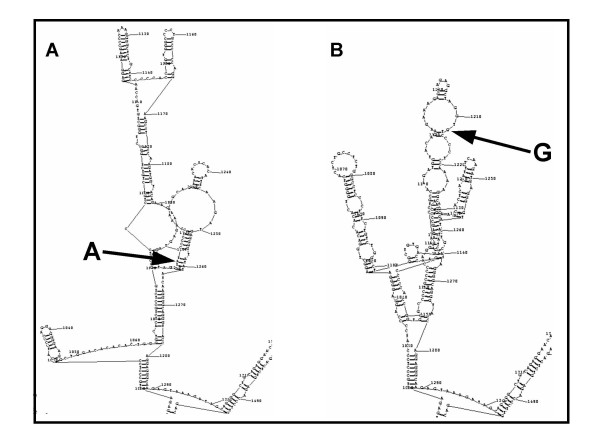
**Mfold analysis of the region surrounding the genetic variant at position 1212 in intron BC of ADAM33**. A) The inclusion of the protective allele A; and B) the inclusion of the at-risk allele G.

Our analysis, using computational tools, of the SNP BC+1 and surrounding DNA sequence has shed some light as to the type of regulatory element that may reside in intron BC, and its importance in regulating ADAM33. Comparative studies with the mouse syntenic region has shown that SNP BC+1 falls within a CNS and is surrounded by several stretches of conserved nucleotide regions that extends all the way into intron AB. This has also been observed in several other vertebrate species [56] and suggests that this region is evolutionarily conserved and has important functional consequences in the maintenance of ADAM33. In addition, the SNP BC+1 region shared significant homology with the NFkappaB p50 sub-unit TF binding site when the protective allele was present. When the at-risk allele was inserted the regulatory element was destroyed. Transcription through the NFkappaB proteins is usually carried out by DNA binding of up to 5 homo or hetero dimers of members of this family [45]. Therefore it is likely that there may be other NFkappaB TF sites in the CNSs that have yet to be identified and that the one centered at SNP BC+1 may require these regulatory elements rather than act on its own. This may also explain that in our *in vitro *model system there was a lack of an increase in transcript expression above the levels of the control (+). Furthermore, the disruption to NFkappaB regulatory elements has been shown to be a trigger for the development of airway diseases [46]. While these computational studies provide some insight as to the effect of a genetic variant on a region of DNA, especially within the ADAM33 gene, further experimental investigation through the use of Electromobility Shift Assays (EMSA) would need to be performed to validate the type of proteins that are recruited to the BC+1 site. We have carried out additional sequence analysis of the region using Mfold [47], which predicts the folding of mRNA. These results would suggest that the change of a single base from A (protective allele) to G (at-risk allele) at nucleotide1212 within intron BC can dramatically alter the folding of the mRNA secondary structure (Fig. 4). These types of affects have been observed in other studies and demonstrate that a single base change at a critical point in a gene can have adverse consequences on mRNA stability, levels of transcription and alternative splice variants [48-53].

## Conclusion

The data presented demonstrate that a disease-associated SNP within ADAM33, an asthma susceptibility gene, does have functional consequences. The mechanistic role of SNP BC+1 appears to be involved in regulating transcription levels. While these enlightening data provide an insight into the role of a disease-associated SNP on the disruption of ADAM33 regulation it is evident that the picture is more complex. SNP BC+1 is a common genetic variant but the at-risk allele appears to have a dramatic modifying effect in our *in vitro *model system. However, *in vivo *this mode of action is likely to be tempered due to the complex interplay of regulatory regions in the processing of the ADAM33 pre-mRNA and may play a role with the promoter in setting the levels of the alternative spliced variants. In order to comprehend the complex interplay of the disease SNPs on ADAM33 gene regulation it will be necessary to identify other regulatory elements. The location of these regions in relationship to disease-associated SNPs would provide the basis for studying their mechanistic role in pre-mRNA processing. Through the use of mini-gene model systems [54] it will be possible to glean a greater understanding of the functional role of these genetic variants to modulate transcription as well as exon splicing in ADAM33. These data, combined with understanding the functional role of the ADAM33 protein in airway remodeling (H. Giese and R. Del Mastro, manuscript in preparation), will lead to unraveling another piece of the asthma puzzle.

## Competing interests

The author(s) declare that they have no competing interests.

## Authors' contributions

RDM participated in the design and co-ordination of the study and drafting of the manuscript; LT generated the plasmids containing the BC+1 genetic variants; HG performed the alkaline phosphatase experiments, TPK and PVE participated in the statistical analysis and study design; KJWM performed the analysis of the sequence surround the BC+1 variant using El Dorado; RDL participated in the study design. All authors have read and approved the manuscript.

## Pre-publication history

The pre-publication history for this paper can be accessed here:


